# Transcriptome Reveals Multi Pigmentation Genes Affecting Dorsoventral Pattern in Avian Body

**DOI:** 10.3389/fcell.2020.560766

**Published:** 2020-10-01

**Authors:** Yang Xi, Hehe Liu, Liang Li, Qian Xu, Yisi Liu, Lei Wang, Shengchao Ma, Jianmei Wang, Lili Bai, Rongping Zhang, Chunchun Han

**Affiliations:** Farm Animal Genetic Resources Exploration and Innovation Key Laboratory of Sichuan Province, College of Animal Science and Technology, Sichuan Agricultural University, Chengdu, China

**Keywords:** transcriptome, plumage color, dorsoventral pattern, avian, molecular regulation

## Abstract

Certain animals exhibit a special dorsoventral pattern with a lighter ventral side compared to the dorsal one and this phenomenon was preserved in the long-term evolution process. Birds also retain this trait. Recently, Inaba et al. (2019) found that *ASIP* (agouti signal protein) regulated interconversion between different melanocyte types leads to dorsal stripe pattern, which may partly explain the birds’ dorsoventral plumage color difference. In this study, we used the embryo samples of LBM (light brown mottling) ducks (*Anas platyrhynchos*) with white ventral and dark dorsal body parts to investigate the mechanism of dorsoventral color variation. Firstly, melanin deposition process of duck embryos was investigated. The result indicated that E13 and E16 were the active stages of melanin synthesis. Moreover, the melanin deposition on the dorsum of LBM ducks was higher than that on the ventral side throughout. Then, RNA-seq was conducted for the dorsal and ventral skin tissues from E7 (early), E13 (middle) and E19 (late) of LBM ducks. Expression pattern analysis showed that the mRNA expression of most melanin synthesis related genes were at the highest level at E13, which was consistent with the section analysis. A correlation was found between melanogenesis pathway and dorsoventral color difference by co-expression analysis. In the DEG (differentially expressed gene) analysis, we added the dorsal skin transcriptome of embryonic white and black duck of same subspecies (*Anas platyrhynchos domestica*) for horizontal comparison. The results showed that 8 melanogenesis related genes (*TYR*, *TYRP1*, *MLANA*, *RAB38*, *OCA2*, *TSPAN10*, *MC1R*, and *MSLN*) were the common DEGs (Differential expressed genes) in the comparisons of body parts and breeds suggesting that the underlying molecular regulatory mechanism of dorsoventral plumage color difference may be similar to that of albino and melanic duck, which were caused by the different expression of multiple genes in melanin synthesis pathway. In addition, the molecular regulation of melanin synthesis pathway in the dorsal and ventral side of LBM ducks was analyzed. In this pathway, *ASIP*, *MC1R*, *TYR*, and *TYRP1* have differential mRNA expression. *ASIP*, as an upstream gene in this pathway, was likely to play a decisive role in determining the dorsoventral plumage pattern.

## Introduction

The diversity of coat color types plays important roles in animal kingdom such as protective colors (Stuart-Fox and Moussalli, 2011), courtship signals (Stuart-Fox and Moussalli, 2008; Stavenga et al., 2011), division of social status (Bergman et al., 2009) and even social communications (Caro et al., 2017). In various coat color patterns of animals, there is a common phenomenon that the pigment deposition of the ventral side has different degrees of dilution compared with that in the dorsum. Scientists considered that the formation of this phenomenon is tend to improve the efficiency of predation since waterfowls or seabirds mainly prey underwater, and lighter ventral plumage can reduce the wearer’s conspicuousness to prey (Cairns, 1986). However, from the perspective of evolution, it is un-precise to explain this complex phenomenon through a single explanation. For example, predators in the sky or on land will not face predatory situations like waterfowls or seabirds. Therefore, it has been speculated that the color difference of dorsal and ventral feathers of birds may be caused by many different selection pressures (Ruxton et al., 2004). At present, some researches have tried to explain this phenomenon in molecular level. In rodents, the ventral specific promoter regulate the *Agouti* gene expression during the hair growth cycle in ventral side leading a lighter ventral hair (Vrieling et al., 1994). In addition, a 216 kb deletion on *TBX15* (*T-box transcription factor 15*) can also control this type of coat color pattern in subtle level (Candille et al., 2004). In birds, it was found that there was a difference in *ASIP* (*Agouti signal protein*) gene expression between dorsal and ventral side in quail and chicken (Nadeau et al., 2008). Studies speculated that the mechanism in chicken might be similar with that in mouse. It’s the specific expression of different spliced variants of *ASIP* in different body parts leading to the difference between dorsal and ventral plumage color (Yoshihara et al., 2012).

The plumage coloration is mainly determined by melanin, carotenoid, and structural color. Structural color is an optical effect formed by the change of light propagation direction caused by some special microstructure arrangement. The most typical example is the greenhead trait of male mallards (Stavenga et al., 2017). Among them, melanin is the most important factor since almost all organisms can synthesize melanin by themselves. The process of melanin deposition is complicated within three main stages which are melanocyte development, pigment production and pigment distribution (Cieslak et al., 2011). Each stage is regulated by its corresponding genes. During the first stage, Melanocytes are derived from melanoblast, which originate from NCCs (neural crest cells). Melanocytes in trunk and limbs are formed by differentiation of trunk NCCs (Bronner and Ledouarin, 2012). NCCs originate from neuroepithelium and enter a migration stagnation area. Then they enter different migration routes and arrive at the exact location of the embryo. The two migration routes aredorsolateral and ventral route (Lin and Fisher, 2007). Before migration begins, a fraction of NCCs are specialized into melanoblasts. When melanoblasts migrate along the dorsolateral route to a designated location, they continue to differentiate into melanocytes and produce melanin (Faas and Rovasio, 1998). After the first stage, it comes to pigment production. The production of melanin depends on melanosomes, a type of lysosomal organelle. The activity of tyrosinase directly determines the content and type of melanin produced (eumelanin/pheomelanin) (Moellmann et al., 1988; Orlow et al., 1993; Mani et al., 2002). Then in the last stage, the melanosome which has synthesized melanin will transport to the outside of melanocyte and deposit in different tissues.

Compared with mammals, bird embryos are easier to collect. In addition, LBM duck, as a new variant in duck breeding process, has more obvious dorsoventral color difference than other duck breeds and even birds. Therefore, the embryonic samples from LBM ducks were taken as the main research objects to figure out which stage of melanin deposition process causes the dorsoventral difference and the molecular regulation mechanism of this phenomenon.

## Materials and Methods

### Animal Samples

The embryo samples from E7 (7-day-old embryos), E10, E13, E16, E19, E22, E25, and D1 (the first day of hatching) of Peking duck (white), Heiwu duck (black) and GF2 duck (light brown mottling) were all from the poultry breeding farm of Sichuan Agricultural University. The ducks are from the same subspecies (*Anas platyrhynchos domestica*) while the breeds are different. Peking duck and Heiwu duck are local breeds in China, and GF2 is a breeding strain which has 100% LBM offspring. Pretreatment was required for collection of section samples and transcriptome sequencing samples. For paraffin section, we would collect 0.5 cm^3^ chunk with muscle and skin in it. In the later stage of embryo development, the well-developed feathers needed to be cut off to retain the complete feather follicle tissue. The samples were stored in 4% paraformaldehyde. For RNA-seq samples, we collected the dorsal skin between the two wings and the ventral skin near the carina (the junction of the chest and abdomen). 1 cm^2^ skin tissue with feather follicles was carefully peeled off with scalpel. The sample needed to be cleaned in PBS (phosphate buffer saline) with 0.1% DEPC (diethyl pyrocarbonate) water. RNA samples are mainly collected from skin carrying intact feather follicles. As with section samples, the extra feathers in later stage of embryo development needed to be cut off as well. At last, the samples were in the RNA-later reagent for storage. All the experimental procedures, described below, were approved by the Animal Ethics Monitoring Committee of Sichuan Agriculture University, and carried out in accordance with Guideline of Animal Welfare China.

### Paraffin Section

After 2-day fixation in 4% paraformaldehyde, the samples were dehydrated, embedded, and sectioned (thickness = 4 μm) for toluidine blue staining. The concentration of toluidine blue dye solution was 1%. The prepared sections were observed and photographed by microscope. The coverage area of melanin was calculated by Image J software (Abramoff et al., 2003). Each treatment group had three biological replicas.

### RNA Extraction

The skin tissues during embryonic development were used as the material to extract total RNA. The skin was put into lysing matrix (MP Biomedicals, LLC) containing 1 mL TRIzol reagent and ground twice for 30 s (oscillation speed: 6 m/s) by tissue crushing apparatus (MP FastPrep-24TM). Total RNA was extracted according to the manufacturer’s protocol. The quality and quantity of RNA samples were checked by Spectrophotometer NanoDrop 2000 and denaturing agarose gel electrophoresis. All RNA samples were treated with DNAse-I (TOYOBO, Shanghai, China) for later use. The RNA integrity number was detected by Agilent Bioanalyzer 2100. The quality and integrity of all samples met the requirements of library construction (Supplementary Table S1).

### RNA-seq

A total amount of 1 μg RNA per sample was used as initial material for the RNA-seq. Briefly, mRNA was purified from total RNA using poly-T oligo-attached magnetic beads, RNA fragmentation and short RNA strands were carried out by NEBNext First Strand Synthesis Reaction Buffer under elevated temperature. Subsequently, First cDNA strand was synthesized using random hexamer primers and RNA fragments as template. Second strand cDNA synthesis was subsequently performed using buffer, dNTPs (dUTP), DNA polymerase I and RNase H. RNA fragmentation and short RNA strands were carried out by NEBNext First Strand Synthesis Reaction Buffer under elevated temperature. The library fragments were purified and elution with EB buffer, then terminal repair, add poly (A) and adapter were implemented. In order to select cDNA fragments of preferentially 400 bp in length, the library fragments were purified with agarose gel electrophoresis and the UNG (Uracil-*N*-Glycosylase) enzyme was used to digest second strand of cDNA. PCR was performed, aimed products were retrieved by agarose gel electrophoresis, and the library was completed. The libraries were clustered and sequenced on an Illumina NovaSeq 6000 platform and 150 bp paired end reads were generated.

### Raw Data Processing

Low quality reads were filtered using stringent criteria by FASTX (v0.0.13): (1) reads with more than 50% of bases with quality < 20; (2) the base quality is < 10 at the 3′ end of the reads; (3) reads with overrepresented adaptors; (4) reads having an ‘N’ base; (5) reads shorter than 20 bp. Hisat2 (v2.1.0) was used to align the clean data to reference genome of Peking duck (IASCAAS_PekingDuck_PBH1.5) (Li et al., 2019). The mapped data was colated and formatted by Samtools. Gffcompare was used to check the assembly of transcripts (Song et al., 2019). Then, Stringtie was used to calculate the gene expression level (Li et al., 2018). At last, packages (genefilter, dplyr, devtools) in R(v3.5.1) were used to analyze DEGs between the experimental group and the control group. The screening criteria of DEGs were *P* < 0.05 and | log_2_FC| > 1.

### Weighted Gene Co-expression Network Analysis (WGCNA)

The “WGCNA” package in R was used to construct the co-expression network for all genes in different groups (Langfelder and Horvath, 2008). Two data tables were required. The first one contained the expression level of all genes calculated from each sample. The second table contained information about the phenotype and developmental stages of each sample. After importing the data tables, the analysis could be started with R.

### Gene Expression Pattern Analysis

The gene expression pattern was analyzed by STEM software (Ernst and Bar-Joseph, 2006). The ‘Log normalize data’ method was adopted in the strategy of expression quantity transformation. Other options were set to default since they have been shown to give optimal results with both biological and simulated data (Ernst et al., 2005). The expression level used in this study was the previously calculated FPKM value. The *P*-value of the clustered profile was less than 0.05, which was considered significant (Xu et al., 2018). By above methods, gene expression patterns of LBM duck’s dorsal and ventral skin were clustered.

### Gene Ontology (GO) and Pathway Enrichment Analysis

DAVID^1^ database was used to conduct GO functional enrichment analysis and generate gene ID of the DEGs among different comparisons (Dennis et al., 2003). IDs of DEGs were used to enrich different signaling pathways. This analysis was conducted using KOBAS (Xie et al., 2011). *Q*-value < 0.05 was considered significant. R language and related packages are used to realize data visualization.

## Results

### Plumage Pattern Comparison of Different Duck Breeds

**FIGURE 1:**
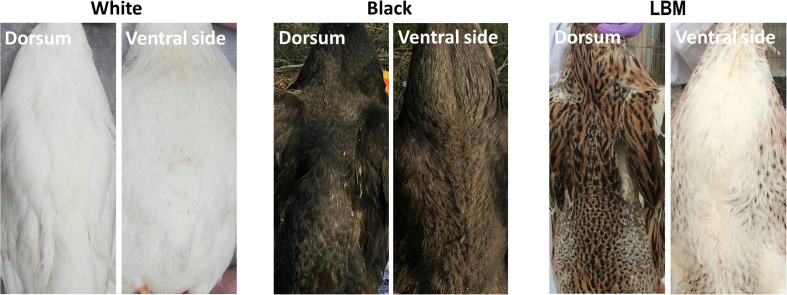
Pigment distribution in adults. The difference of pigment deposition between the dorsum and ventral side of white, black, and LBM ducks.

We compared several common duck feather color breeds (white, black, and LBM) of the adult stage. The whole body pigment distribution of black ducks is evenly distributed. In white ducks, there is no pigment deposition in the whole body. However, the ventral side of the LBM ducks, including the chest, turned white while the dorsal part of the duck still had obvious pigment deposition (Figure 1). LBM ducks have typical and obvious dorsoventral difference of pigment deposition. It should also be noted that the skin color of all three breeds is white.

**FIGURE 2:**
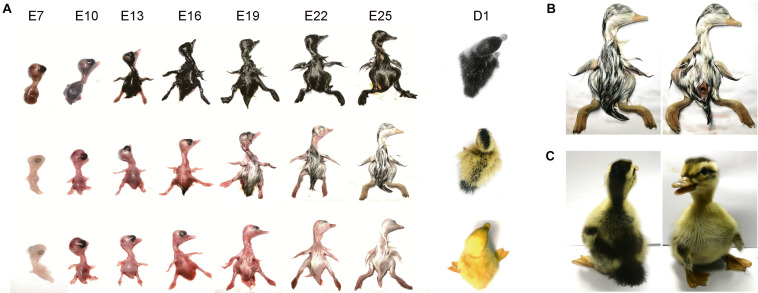
Pigment distribution in embryos. **(A)** Changes of plumage color phenotype during embryonic development. From left to right represent different stages of embryonic development; from top to bottom represent black, LBM and white duck. **(B)** Differences of dorsum and ventral side plumage color in LBM embryos. **(C)** Differences of dorsum and ventral side plumage color in LBM ducks after hatch.

Similar comparisons were made with samples from the embryonic development stage as well (Figure 2A). The visible skin melanin deposition was observed in E13 stage of LBM ducks, while it was earlier in the black ducks, the melanin production of which could be observed in the dorsum during E7. With the development of plumage, there was no significant change in pigment parts of these three duck embryos. However, similar with the adult ducks, there were obvious differences between dorsum and ventral side of LBM ducks (Figures 2B,C).

**FIGURE 3:**
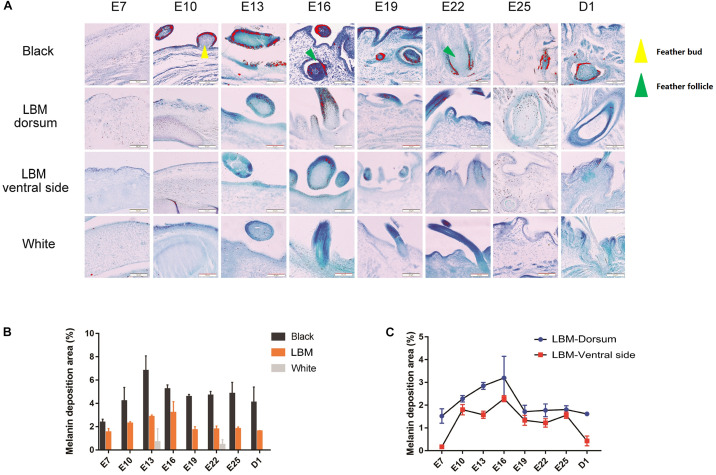
Melanin deposition area analysis. **(A)** Observation of melanin in different breeds and different body parts of LBM embryos. Red particles = visible melanin; Yellow triangle = feather bud; Red triangle = feather follicle. **(B)** The amount of melanin deposition per unit area in different dorsal skin tissues. **(C)** The amount of melanin deposition per unit area in dorsal and ventral skin tissues in LBM ducks.

Then we collected dorsal skin samples of LBM, white and black duck with days 7, 10, 13, 16, 19, 22, 25 of embryo (E7–E25) and the 1st day after hatching (D1) for section making and toluidine blue staining to conduct further verification of phenotypic differences (Figure 3A). We could see that before E16, there was no fully developed feather follicle tissue. Only the feather buds was observed. Moreover, melanin (red particles) is mainly deposited in the feather buds and hair follicles at different stages. There is little melanin deposition in the skin or other tissues. The melanin deposition area was calculated and compared throughout different developmental stages, breeds and body parts. At the developmental stage level, melanin content increase constantly during the E7–E13 of LBM and black ducks. After a period of rapid increase, the melanin deposition area in the dorsum began to decrease and tend to a stable level. In the comparison of different breeds, there was almost no melanin can be observed in the dorsal skin of white ducks. Meanwhile, the melanin content of black embryos was higher than that of LBM embryos at every stage (Figure 3B). In addition, we analyzed the melanin deposition area in dorsal and ventral skin of the LBM ducks and found that the melanin content in the ventral skin was always lower than that in the dorsum at every embryonic stage (Figure 3C).

### Overview of Transcriptome Sequencing

**FIGURE 4:**
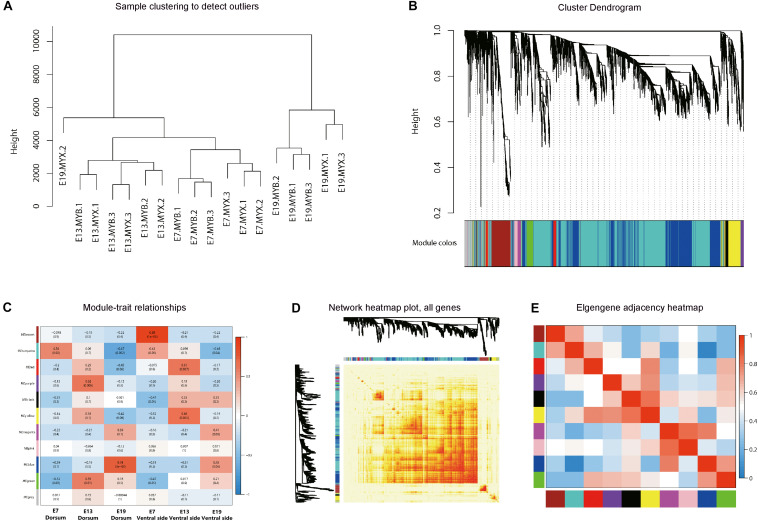
Weighted gene co-expression network analysis (WGCNA). **(A)** Sample clustering diagram. E + number represents different embryonic development stages. MYB and MYX represents dorsal and ventral skin samples of LBM embryo, respectively. **(B)** Hierarchical clustering tree. Different colors on the abscissa represent different clustering modules. **(C)** Correlation between modules and traits. The abscissa represents different trait groups, and the ordinate represents different modules. **(D)** Visualized network heat map. **(E)** Correlation diagrams between modules. The redder the color of the area where different modules intersect is, the stronger the correlation.

Three embryonic stages of E7, E13, and E19 from the dorsal and ventral skin tissues of LBM ducks were selected for RNA-seq (triplicate biological replicas in each group). We analyzed the skin and feather follicles together since it is difficult to separate the feather follicles from the skin during the embryonic stage. It was found that there was no pigment deposition in the skin of the three kinds of ducks, indicating that skin tissue had little effect on the expression of genes related to pigment synthesis in follicles. The quality and mapping rate of all samples were shown in Supplementary Tables S1, S2. First, we conducted the co-expression analysis of all genes from the samples of different developmental stages of LBM embryos. The 18 samples were divided into two categories which were the samples in E19 and samples in E7 and E13. Under these two categories, the gene expression patterns of the dorsum and ventral side were also separated (Figure 4A). Then, the genes with similar expression patterns were classified into different modules (Figure 4B). The correlation analysis between the modules and phenotypes showed that the blue module had a strong correlation between the dorsum and ventral side in E19 stage (Figure 4C). KEGG analysis was conducted to find out the main pathways in which the genes in blue module were included, and the top30 pathways were screened out by *Q*-value. The melanogenesis pathway was found to be involved (Supplementary Table S3). Meanwhile, the correlation analysis between modules was conducted as well and the results indicated that the gene expression pattern in blue module was similar to that in purple module (Figures 4D,E).

### The Gene Expression Profile Clusters During Embryonic Development of Dorsal and Ventral Skin

**FIGURE 5:**
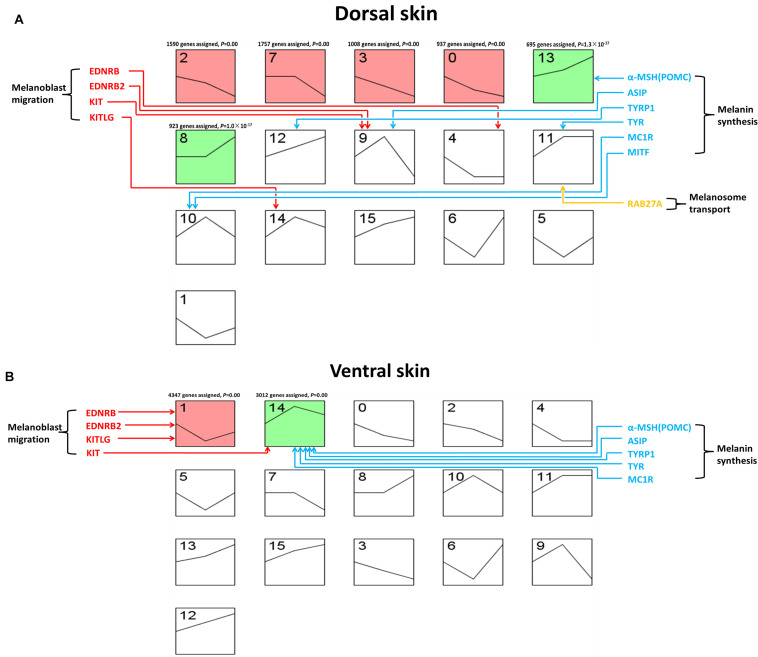
Gene expression pattern of LBM embryonic development stage. The horizontal axis of each block represents the developmental stage. The three developmental stages from left to right are E7, E13, and E19. The vertical axis represents the gene expression level. Colored boxes indicate that the *P*-value is less than 0.05. Boxes with the same color indicate that their expression patterns are similar. **(A)** Clustering of expression patterns of genes in the dorsal skin development of LBM embryos. **(B)** Clustering of expression patterns of genes in the ventral skin development of LBM embryos.

The analysis of gene expression profiles in different stages could prove a better understanding to the development process of the embryonic development. Therefore, we used STEM (Short Time-series Expression Miner) software to analyze the gene expression profile of skin transcriptome in the dorsum and ventral side of LBM ducks. In dorsal skin, all genes were divided into 16 clusters (0–15) and six clusters with significance were clusters 2, 7, 3, 0, 13, and 8. Among the six clusters, the expression profiles of clusters 2, 7, 3, and 0 were similar and the gene expression decreased gradually from E7 to E19. The expression profile of the other two similar clusters 13 and 8, was gradually increased with the development of embryo (Figure 5A). Similarly, we also get 16 clusters while only two clusters (1, 14) were significant in ventral skin. Meanwhile, the expression profiles of these two clusters were totally different. It could be seen that in cluster 1, the gene expression level first fell in E13 and then rose in E19. That in cluster 14 was diametrically opposite. It first rose and then fell (Figure 5B). Then, the KEGG (Kyoto Encyclopedia of Genes and Genomes) enrichment analysis of genes from each significant cluster was conducted. We demonstrated the top 15 pathways in each cluster (Supplementary Table S4). In the ventral skin, the melanogenesis pathway was enriched in cluster 14 within many development related pathways such as WNT, MAPK, m-TOR and notch signaling pathways. Meanwhile, melanogenesis pathway was not enriched in any significant clusters of dorsal skin.

Moreover, we investigated the expression profile of the marker genes related to different melanin deposition processes. The genes related to the melanoblast migration were enriched in clusters 4, 9, and 14. The expression profile of 9 and 14 was similar which increased first and then decreased. The result was different for the melanoblast migration related genes in ventral skin group. They were mainly enriched in clusters 1 and 14, most of them were in cluster1 which first decreased then increased. The expression profile of melanin synthesis related genes was also different in the skin of dorsum and ventral side. The genes controlling melanin synthesis were enriched in clusters 13, 12, 9, 11, and 10 in dorsum while those in ventral skin were all enriched in cluster 14. As we mentioned before, the trend of cluster 14 first rose and then declined which is similar to cluster 9 and cluster 10. However, the mRNA expression profile of *POMC* (*proopiomelanocortin*), *TYR* (*tyrosinase*), and *TYRP1* (*tyrosinase-related protein 1*) in clusters 13, 12, and 11 kept rising all the time. As for the genes related to melanosome transport, only *RAB27A* (*member RAS oncogene family*) was enriched in dorsal cluster 12 which was on the rising trend.

### Differential Expressed Gene Analysis

In order to fully understand the formation of different plumage color between dorsal and ventral side, we added two other plumage color groups which were white and black for horizontal comparison.

**FIGURE 6:**
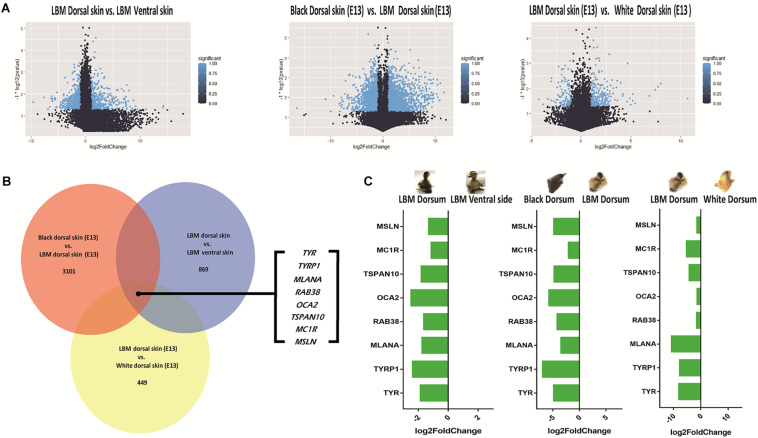
DEGs screening. LBM is the abbreviation for light brown mottling. **(A)** Volcano map of DEGs in different compared groups. **(B)** The common genes included in different comparisons. **(C)** The tendency of differential expression.

In the comparison between dorsal and ventral skin of LBM embryos, a total of 869 DEGs (Differential expressed genes) were screened out under the criteria of | log2FC| > 1 and *P* < 0.05. Under the same criteria, 3101 (black vs. LBM) and 449 (LBM vs. white) DEGs were screened out respectively by comparing different plumage color breeds. The results were shown in the volcanic plot graphs (Figure 6A). Then we analyzed the common DEGs in all comparisons. The intersection contains only eight coding genes which were *TYR*, *TYRP1*, *MLANA* (*melan-A*), *RAB38* (*member RAS oncogene family*), *OCA2* (*OCA2 melanosomal transmembrane protein*), *TSPAN10* (*tetraspanin 10*), *MC1R* (*melanocortin 1 receptor*) and *MSLN* (*mesothelin*) (Figure 6B). The expression level of these genes in the dorsal skin was all relatively high compared with that in ventral skin (Figure 6C) suggesting that they might have similar functions.

### Molecular Regulation Mechanism of Melanin Synthesis Difference in Dorsolateral Side

**FIGURE 7:**
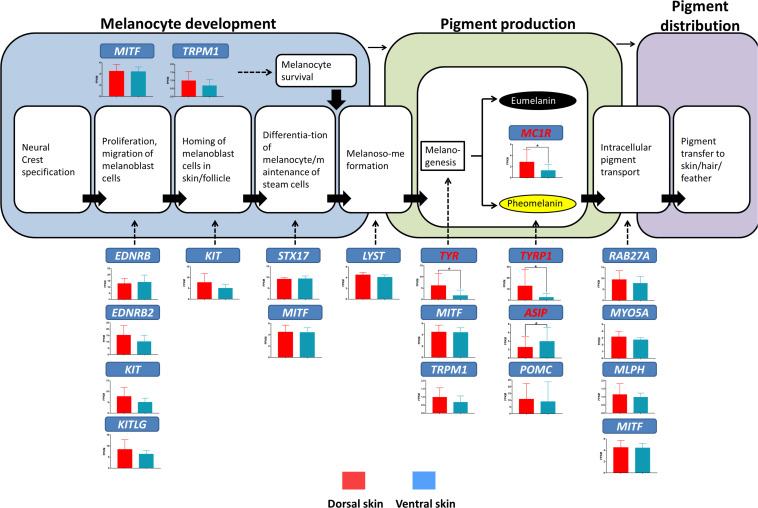
Gene expression levels at different stages of melanin production in LBM ducks (Cieslak et al., 2011). The bar charts show the mRNA expression of genes responsible for different melanin deposition processes in the dorsal and ventral skin. The red bars represent the mRNA expression level in dorsal skin; the blue ones represent that in ventral skin. Red marked genes are those that show significantly different mRNA expression level between the dorsum and ventral side.

The above result suggested that the plumage color differences in the dorsum and ventral side of the LBM ducks may be caused by the gene expression differences related to melanin synthesis. For further verification, we investigated the expression level of marker genes responsible for different melanin deposition processes in the dorsoventral skin of LBM ducks (Figure 7). It could be seen from the results that *TYR* (*P* = 0.01), *TYRP1* (*P* = 0.02), *ASIP* (*P* = 0.04), and *MC1R* (*P* = 0.02), marked genes in melanin synthesis stage, were differential expressed. Except for the 4 genes mentioned above, there was no significant difference in the mRNA expression of other genes. It is worth noting that the genes with different expression in the comparison in the two body parts were almost highly expressed in the dorsum while *ASIP* gene was opposite. Its expression level in ventral skin was higher than that in dorsal skin.

## Discussion

### The Embryonic Melanin Deposition Process of LBM Ducks

From the paraffin section results, the melanin per unit area in E13 and E16 were detected at the highest level compared to other stages. We speculated that the period from E13 to E16 was the active stage of melanin synthesis. The expression pattern analysis demonstrated this view as well, especially in the ventral skin. Most of melanin synthesis related genes showed a high expression in E13. But in the dorsum, some genes related to melanin synthesis kept rising all the time including *TYR*, *TYRP1*, and *α-MSH* (*POMC*). Among them, TYR and TYRP1, as the key rate limiting enzyme in melanin synthesis, can indirectly reflect the melanin production content (Moellmann et al., 1988; Orlow et al., 1993; Mani et al., 2002; Westerhof, 2006). The results indicated that the melanin synthesis of the dorsal skin was more active than that of the ventral skin in the later stage of embryo development. This also explains why the expression level of *RAB27A*, a melanosome transport related gene, increases in dorsal skin with the development of embryo. The more active melanin synthesis is, the more melanosomes need to be transported outwards (Bahadoran et al., 2001). In addition, we also found that there was a dorsoventral difference in the expression profile of genes related to the migration of melanoblasts of early middle embryonic development (E7–E13). The migration of melanoblasts in all vertebrates starts from the dorsal side. Therefore, from the gene expression profile in the dorsal skin, we can infer that the migration process of melanoblasts in the LBM ducks has been basically completed in E13 stage since most of the related genes began to decline after this stage. However, it is difficult to judge whether the migration process has a decisive effect on the final dorsoventral pigment deposition from the trend of gene expression. Because at the end of embryonic development, the mRNA expression of most genes related to melanoblasts increased again.

### The Melanin Synthesis Related Genes Is the Main Cause of Dorsoventral Plumage Color Difference

There are obvious coloration differences between the dorsal and the ventral plumage of adult LBM ducks. Our research confirmed that such difference has appeared as early as the embryonic stage. Through the results of paraffin section, we found that there were differences in melanin content between the dorsal and ventral skin tissues of the LBM embryos.

As shown in the result of DEGs screening, eight DEGs were screened out in the intersection of three comparison groups. Among them, *TYR*, *TYRP1*, and *MC1R*, as major candidate genes for melanin synthesis, were held within the 8 genes. It has been reported that a 6.6 kb insertion on the intron of *MITF* (*melanocyte inducing transcription factor*) resulted in the failure of expression of *MITF-M* spliced variant (Zhou et al., 2018). This spliced variant is specially responsible for the regulation of melanin production and it can activate tyrosinase (Shibahara et al., 2000). *MC1R* is another key gene for melanin synthesis, and its mutations have been found to have significant association with the extended black trait of ducks (Yu et al., 2013). In addition to the well-known candidate genes mentioned above, MLANA, RAB38, OCA2, TSPAN10, and MSLN were also reported to incorporate into the process of melanin synthesis (Lu et al., 2010; Coppola et al., 2016; Seberg et al., 2017; Béziers et al., 2019). The result showed that the difference on the dorsum and ventral side of LBM ducks might be caused by the mRNA expression changes of multi melanin synthesis related genes.

In addition, it is also necessary to consider whether genes related to melanocyte development can cause this phenotype. In our opinion, the possibility is relatively low. First, only *TYR*, *TYRP1*, and *MC1R* were found to have significant differential expression after analyzing the expression level of marker genes related to melanocyte development, melanin synthesis and melanosome transport. Second, from the phenotypic characteristics, the plumage patterns caused by melanocyte development related genes often have obvious borders between the non- and colored areas (Miwa et al., 2007; Hauswirth et al., 2013; Li et al., 2015; Xi et al., 2020). The loss of function of these genes will lead to the complete absence of melanocytes in some body parts, resulting in pure white spots while the plumage color transition from the dorsum to the ventral side of LBM ducks is a gradual process, and there is no obvious boundary. Meanwhile, our section results showed that melanin was deposited in the dorsum of LBM ducks supporting that melanoblasts did migrate to the ventral side and differentiate normally to produce pigment. Therefore, we speculated that the dorsoventral difference of plumage color of LBM ducks was mainly caused by the differential expression of melanin synthesis related genes.

### Melanin-Mediated Mechanism of Dorsoventral Plumage Color Formation

**FIGURE 8:**
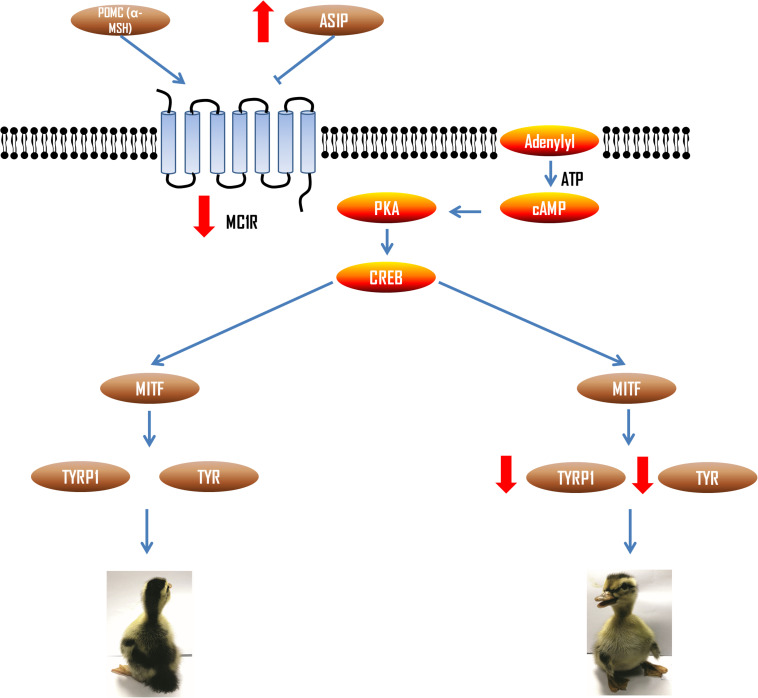
Regulation of melanin synthesis pathway related genes in dorsal and ventral skin of LBM ducks. Red arrows represent the expression level change of each gene in ventral skin of LBM duck.

*TYR* and *TYRP1* are the terminal genes of melanin synthesis process, which are mainly regulated by cAMP mediated signal. In this signal transduction pathway, upstream agonist *α-MSH* (*POMC*) and inhibitor *ASIP* can competitively bind to *MC1R* to increase and decrease cAMP concentration, so as to regulate *TYR* and *TYRP1* expression (Figure 8). We found that the mRNA expression of *ASIP* was higher in the ventral side. ASIP is an inhibitor ligand of MC1R. Its binding with MC1R receptor can reduce cAMP content to inhibit signal transduction and thus reduce tyrosinase activity (Suzuki et al., 1997). Therefore, the results suggested that the MC1R receptor binds with more ASIP in the ventral skin of the LBM ducks, which resulted in the decrease of expression of the downstream genes such as *MITF*, *TYR*, and *TYRP1*. In addition, *MITF* can also reverse regulate the expression of *MC1R* (Aoki and Moro, 2002), which is the reason why the *MC1R* gene expression itself was also increasing in the dorsum compared with ventral side. It is worth noting that *ASIP* has also been shown to have a dorsoventral differential expression level in quail and chicken (Nadeau et al., 2008). It suggests that this molecular regulation model may also be applicable to other birds.

### Cell Fate May Affect the Dilution of Ventral Plumage

Dahmann and Basler have proposed a model for the formation of compartment boundary. When stem cells reach the compartment boundary, they are faced with differentiation or gene expression (Dahmann and Basler, 1999). Melanocytes initially come from NCCs which are with multiple differentiation potential in the process of embryonic development. In the analysis of gene expression patterns of ventral transcriptome, we found that the melanogenesis pathway and multiple growth-related signaling pathways including WNT, MAPK, m-TOR, and Notch were enriched in the same cluster (cluster 14). This provides us with a conjecture that when NCCs reach the compartment boundary during migration process, they will start to choose to differentiate into other cells to play their functions and thus reduce the number of melanocytes. For example, sternum formation regulated by wnt and notch pathways may be one of the factors (Baron and Rawadi, 2007; Ma et al., 2015). Chondrocytes are also differentiated from NCCs. The formation of lateral somitic frontier in chicken has been widely studied (Sudo et al., 2001). In addition, we considered that *ASIP* expression may be one of the important factors affecting the above process since the above process is bound to be accompanied by changes in the stem cell niche in dorsal and ventral feather follicles. The expression of *ASIP* has been shown to affect the stem cell niche features, resulting in variable plumage color (Lin et al., 2013). Recent studies have found that a spontaneous and periodic transformation of avian melanocyte (interconversion between eu- and pheomelanocyte) can take place by *ASIP* regulation, leading special plumage pattern such as the stripe pattern of Japanese quail (Inaba et al., 2019). In view of the complexity of bird plumage color regulation, this model may also explain the formation of plumage color of LBM duck since some small spots or stripes can be observed on the dorsal part of LBM ducklings. However, similar traits do not exist in most bird species. In contrast, the dorsoventral difference of plumage or coat color is more common in birds or even mammals. We consider that the cell fate determination model may be able to explain this phenomenon in a larger scope.

## Data Availability Statement

The authors acknowledge that the data presented in this study must be deposited and made publicly available in an acceptable repository, prior to publication. Frontiers cannot accept a manuscript that does not adhere to our open data policies.

## Ethics Statement

The animal study was reviewed and approved by Animal Ethics Monitoring Committee of Sichuan Agriculture University. Written informed consent was obtained from the owners for the participation of their animals in this study.

## Author Contributions

YX and LW performed the data analysis of transcriptome. YX, SM, and YL performed the section experiment. YX, HL, QX, JW, CH, LB, and RZ completed the manuscript writing. LL participated in the writing instruction and revision of the manuscript. All authors have read and approved the manuscript.

## Conflict of Interest

The authors declare that the research was conducted in the absence of any commercial or financial relationships that could be construed as a potential conflict of interest.

## Footnotes

1 https://david.ncifcrf.gov/

## Supplementary Material

TABLE S1 Sample quality control.

TABLE S2 The reads account and mapping rate of samples.

TABLE S3 KEGG analysis of the genes in blue module.

TABLE S4 Top 10 KEGG pathways in different clusters of gene expression pattern.
